# Outcomes During Intended Fluoroscopy-free Ablation in Adults and Children

**DOI:** 10.19102/icrm.2018.090904

**Published:** 2018-09-15

**Authors:** Ryan T. Kipp, Jason R. Boynton, Michael E. Field, Jesse F. Wang, Anton Bares, Miguel A. Leal, Nicholas H. Von Bergen, Lee L. Eckhardt

**Affiliations:** ^1^Electrophysiology Service, Division of Cardiovascular Medicine, Department of Medicine, University of Wisconsin Children’s Hospital, Madison, WI, USA; ^2^University of Wisconsin Hospitals and Clinics, Madison, WI, USA; ^3^Pediatric Electrophysiology, Department of Pediatrics, University of Wisconsin Children’s Hospital, Madison, WI, USA

**Keywords:** Ablation, arrhythmia, electroanatomic mapping, fluoroscopy

## Abstract

Electroanatomic mapping (EAM) systems facilitate the elimination of fluoroscopy during electrophysiologic (EP) studies and ablations. The rate and predictors of fluoroscopy requirements while attempting fluoroscopy-free (FF) ablations are unclear. This study aimed (1) to investigate the rates of fluoroscopic use and acute success in patients initially referred for FF ablation and (2) to identify procedural characteristics associated with fluoroscopic use in patients in whom FF ablation was initially planned (IFF). We performed a retrospective review of all patients who underwent IFF EP study or ablation between 2010 and 2013. Patient and procedural characteristics were compared between those with successful FF procedures and those who subsequently required fluoroscopy during their procedure. An FF EP study with or without ablation was performed in 124 patients during 138 procedures for either supraventricular or idiopathic ventricular arrhythmias. Of the 138 procedures, 105 of them were performed without fluoroscopy. In the remaining 33 cases, fluoroscopy was used for an average of 1.21 minutes ± 1.18 minutes. Acute procedural success was achieved in 97% of both FF and fluoroscopy procedures. The primary reason for fluoroscopy use was as a guide for transseptal puncture. There were no significant differences between FF and fluoroscopy procedures with respect to catheter placement time or complication rate. In conclusion, in this single-center study of IFF procedures, despite careful case selection for IFF ablation, 24% of IFF cases ultimately required minimal fluoroscopy. Fluoroscopy and FF procedures had similar rates of procedural success and complications. Additional large prospective studies are required to further investigate the safety and efficacy of FF ablations.

## Introduction

The cumulative effect of ionizing radiation exposure from fluoroscopy used for percutaneous interventional procedures has become an increasingly important topic for the electrophysiologic (EP) community and patients. Ionizing radiation puts patients and staff at risk of deterministic and stochastic effects, including malignancy, radiation skin injury, operator cataract, and genetic defects, with pediatric patients being particularly vulnerable to even small doses of radiation.^1^ Standard protective lead aprons worn by physicians and staff leave areas exposed and have led to cancers of the bone marrow and brain.^2^ Additionally, the use of lead aprons places operators and staff at risk of occupational orthopedic injury.^3^

Three-dimensional (3D) electroanatomic mapping (EAM) systems have become useful imaging adjuncts to guide catheter movement, and recent advances in the resolution of imaging now enable catheter manipulation to occur with millimeter-level precision.^4,5^ These systems allow for the enhanced definition of structures poorly visualized with fluoroscopy and the 3D reconstruction of arrhythmia circuits and ablation points. With improved precision in anatomical resolution with EAM systems, the use of either very little or no fluoroscopy have become viable options for EP studies and ablations. The aim of the present investigation was to compare the rates of fluoroscopic use and the outcomes of patients referred with the intention to receive a fluoroscopy-free (FF) (IFF) procedure.

## Methods

### Patient selection

We conducted a retrospective analysis of all of the EP studies completed between February 2010 and August 2013 at the University of Wisconsin Hospitals and Clinics. Cases were included in the present study if the procedure performed was intended to be performed in a FF manner by the attending physicians (L. E. and N. V. B.). Children were defined as individuals who were younger than 18 years of age. Patients were excluded if, during preprocedural documentation, IFF protocol was considered inappropriate by the attending person performing the ablation procedure due to the anticipated need for fluoroscopy. Such cases included those with the presence of ventricular tachycardia in myopathic ventricles; an obvious need for transseptal puncture; or an increased probability of an epicardial arrhythmic focus (eg, arrhythmogenic right ventricular cardiomyopathy). This study was reviewed and approved by the institutional review board (IRB) at the University of Wisconsin–Madison (IRB no. 2013-0469). All patients involved consented to undergo the use of minimal to no fluoroscopy during their procedure. All antiarrhythmic medications were stopped at least five half-lives prior to the procedure. No patient underwent preprocedural magnetic resonance imaging or computed tomography scans in preparation for the procedure.

Patient and procedure characteristics were collected via a review of electronic health records, intracardiac tracings, procedure logs, and procedure reports. Collected data included patient characteristics, acute procedural success rates, complications, time to catheter placement, fluoroscopy time, and dose of fluoroscopy used.

### Procedure description

**Figure 1:**
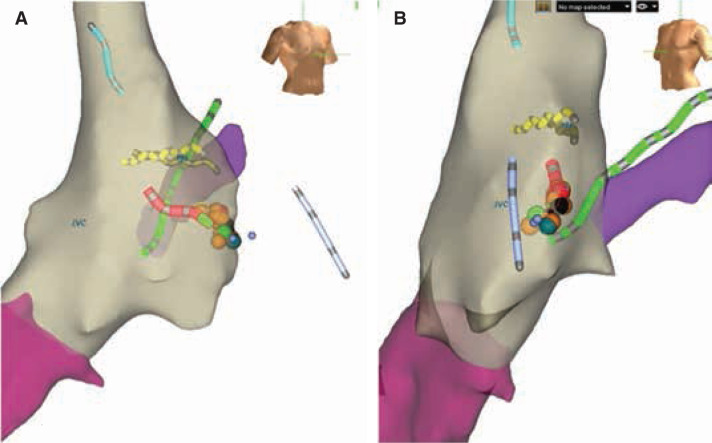
Representative 3D EAM image from an FF ablation for AV nodal reentry tachycardia. The right anterior oblique **(A)** and left anterior oblique **(B)** show right atrial (tan), coronary sinus (purple), and inferior vena cava (fuschia) reconstruction and respective diagnostic catheters (right atrial in light blue, right ventricle in periwinkle, coronary sinus in green, and His bundle in yellow). The cryoablation catheter is shown in red, with ablation lesions marked by circles.

All procedures were performed in the EP laboratory and involved attending electrophysiologists with or without electrophysiology fellows. The EnSite™ Velocity™ EAM system with EnSite™ NavX™ technology (Abbott Laboratories, Chicago, IL, USA) was used for 3D mapping in all cases **(Figure 1)**. The BARD EP recording system (Boston Scientific, Natick, MA, USA) was used for intracardiac recordings. Monoplane or biplane fluoroscopy (Philips Healthcare, Andover, MA, USA) could immediately be activated if it was felt that additional imaging was needed. Conscious sedation and local anesthetic were used for all adult patients and general anesthesia was used for all pediatric patients. A bifemoral approach was used in all cases. Intracardiac echocardiography was employed at the discretion of the attending electrophysiologist.

### Follow-up

All patients were evaluated in the outpatient clinic at four weeks to six weeks postoperatively. Additional cardiac monitoring was performed based on symptom presence or clinical suspicion for arrhythmia recurrence. Complications were tracked at the postprocedural visit, in the patient’s medical record, and at subsequent visits to the clinic.

### Definitions

Time to catheter placement was defined as the time of first diagnostic catheter insertion to the time of baseline measurements or to the time that electrophysiology pacing maneuvers or activation mapping were started, whichever occurred first. Placement time was excluded if intracardiac recording information was corrupted or missing, or if an arrhythmia occurred spontaneously before all diagnostic catheters could be placed.

Acute procedural success was considered to be achieved when the clinical arrhythmia could not be induced after the ablation. The waiting period was left to the discretion of the attending physician.

Those patients for whom an IFF ablation was intended, but in whom, according to the operator’s intraprocedural discretion, any degree of fluoroscopy was performed during any part of the procedure, were considered to have crossed over into the fluoroscopy-use group. For fluoroscopy-requiring patients, the reason for fluoroscopy was documented in the procedure notes and verified by reviewing the recording system notes.

Major complications were defined as cardiac or vascular perforation, major bleeding (requiring blood transfusion), persistent atrioventricular (AV) block, a need for permanent pacing, tamponade, stroke, myocardial infarction, or death. Minor complications were defined as transient AV block or prolongation of the A–H interval and bleeding not requiring blood transfusion.

### Statistical analysis

Continuous variables are expressed in the formats of mean ± standard deviation and/or range, as appropriate. Categorical data are expressed as numbers. Obesity determinations for individuals aged younger than 20 years were calculated using the United States Centers for Disease Control and Prevention (CDC) percentile method. For individuals aged ≥ 20 years, a body mass index of 30 kg/m^2^ or higher was considered to indicate obesity, in accordance with the CDC guidelines for adults and children.

Statistical analysis was performed to examine differences and correlations in groups, patient characteristics, and diagnostic catheter placement times. Fisher’s exact test was used to examine differences in the FF and fluoroscopy-use groups as they related to comorbidities. A two-tailed t-test was used to compare FF and fluoroscopy-use transseptal frequency and diagnostic catheter placement times.

## Results

### Patient characteristics

**Figure 2:**
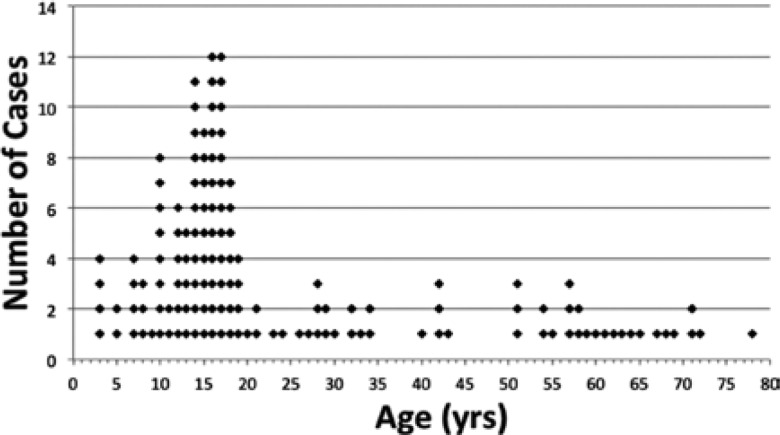
Age distribution of all of the patients included in this analysis.

**Figure 3:**
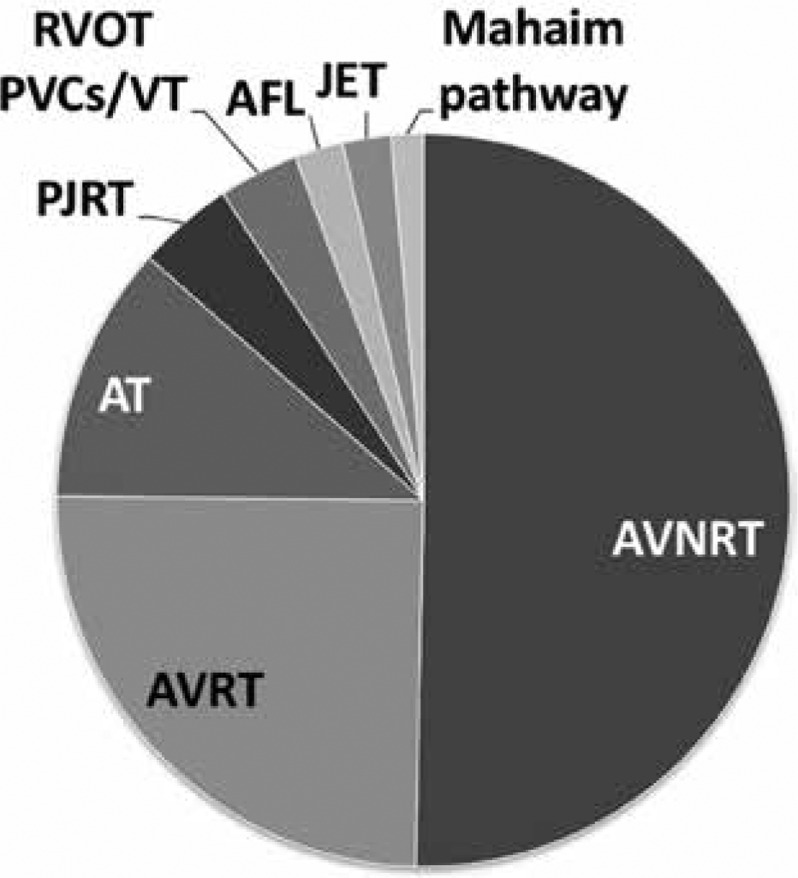
Diagnosis distribution of all patients. In this study, there were 71 patients with AV nodal reentry tachycardia, 35 with AV reentry tachycardia, 16 with atrial tachycardia, six with persistent junctional reciprocating tachycardia, five with right ventricular outflow tract premature ventricular complexes/ventricular tachycardia, three with atrial flutter, three with junctional ectopic tachycardia, and two with Mahaim pathway. AFL: atrial flutter; AT: atrial tachycardia; AVNRT: atrioventricular nodal reentry tachycardia; AVRT: atrioventricular reentry tachycardia; JET: junctional ectopic tachycardia; PJRT: persistent junctional reciprocating tachycardia; RVOT PVCs/VT: right ventricular outflow tract premature ventricular complexes/ventricular tachycardia.

A total of 138 IFF cases for EP study with or without ablation in 124 unique patients from February 2010 to August 2013 were included for analysis. Patient characteristics are outlined in **Table 1** and age distribution is plotted in **Figure 2**. The patients with inducible arrhythmia(s) diagnosed and ablated are shown in **Figure 3** and include those with AV nodal reentry tachycardia (n = 71); AV reentry tachycardia (n = 35); atrial tachycardia (n = 16); persistent junctional reciprocating tachycardia (n = 6); right ventricular outflow tract premature ventricular complexes/ventricular tachycardia (n = 5); atrial flutter (n = 3); junctional ectopic tachycardia (n = 3); and Mahaim pathway (n = 2). Out of 124 patients, eight underwent two ablation procedures and three underwent three procedures (either for the original arrhythmia or a separate arrhythmia). Another three patients also had two separate arrhythmia mechanisms identified for a total of 141 different arrhythmias and 138 IFF cases.

### Procedural characteristics and the use of fluoroscopy

All patients included in this analysis underwent the procedure with the initial intention of completing FF EP study and ablation. Use of fluoroscopy occurred in 33 out of 138 cases (24%). The only significant difference between the characteristics of the patients demonstrating FF versus fluoroscopy-use status requiring ablation was the presence of congenital heart disease (p = 0.038), with fluoroscopy being less likely to occur in cases with congenital heart disease. No other significant statistical differences or strong correlations were found.

In the fluoroscopy group, the total fluoroscopic time ranged from 0.02 minutes to 4.08 minutes, with an average of 1.21 minutes ± 1.18 minutes. The total fluoroscopic dose ranged from 0.11 mGy to 43.01 mGy. The most common reason for fluoroscopy use was transseptal puncture, which accounted for 18 of the 33 fluoroscopy events. While not all transseptal cases required fluoroscopy (12 of the 30 procedures in this series requiring transseptal puncture were done without fluoroscopy), transseptal puncture cases were significantly more likely to use fluoroscopy as compared with were those without transseptal puncture (p = 0.0001). Other reasons for the use of fluoroscopy were to verify venous rather than arterial placement during the placement of large sheaths (those ≥ 11 on the French scale; n = unknown), to assist with stable diagnostic catheter placement (n = unknown), or for ablation catheter positioning (n = 15). Baseline characteristics and arrhythmia diagnosis for the fluoroscopy group are detailed in **Table 2**. Patients requiring transseptal puncture required significantly more fluoroscopy (1.95 minutes ± 1.13 minutes, 30.18 mGy ± 17.38 mGy) than did those without transseptal puncture (0.35 minutes ± 0.28 minutes, 5.75 mGy ± 14.44 mGy; p < 0.0001).

The time to catheter placement was obtained for 89 of the 105 FF procedures (95%) and 22 of the 33 fluoroscopy-use procedures (67%). There was no significant difference in catheter placement time between the FF and fluoroscopy-use procedures (FF: 11.32 minutes ± 8.82 minutes versus fluoroscopy-use: 11.00 minutes ± 5.33 minutes; p > 0.05).

Of the 138 total procedures, 135 proceeded with ablation, while the remaining three were EP studies only. Acute procedural success occurred in 131 of the ablation cases (97%). There was no significant difference in acute procedural success between FF and fluoroscopy-use procedures (acute success for FF occurred in 101 of 104 procedures versus in 30 of 31 fluoroscopy-use procedures). Acute success did not occur in two patients with AV reentry tachycardia (both with para-Hisian pathways with transient AV block during cryoablation) and in two patients with right ventricular outflow tract premature ventricular complexes. Ninety-seven procedures used only cryoablation, 28 procedures used only radiofrequency ablation, and 10 procedures used both energy sources.

Eleven of the 135 cases (8.1%) required more than one procedure due to arrhythmia recurrence following the original ablation: eight patients underwent two procedures and three patients underwent three procedures **(Table 3)**. There was no significant difference in arrhythmia recurrence following FF and fluoroscopy-use ablation procedures.

### Complications

There were no major complications in the study. Minor/transient complications occurred during four FF ablations (3.81%). One patient developed transient AH prolongation; two patients demonstrated transient AV block, which recovered following ablation cessation; and one patient developed a clinically significant hematoma requiring prolonged bed rest. A minor/transient complication (transient AV block) also occurred during one ablation with fluoroscopy (3.03%).

## Discussion

In this cohort of adult and pediatric patients, we demonstrate that FF ablation has a high procedural success rate and a low risk of complications, though minimal fluoroscopy use may occur, especially if left atrial access is required. However, even when left atrial access was required, 40% of transseptal cases could still be completed in an FF manner. Safe elimination of fluoroscopy represents a valuable and accessible tool toward the ongoing endeavor to minimize procedural radiation exposure. The techniques presented here have been pivotal in patient care at our institution and FF and fluoroscopy-reduced procedures have become a new standard.

We determined that a high percentage of patients with a wide range of arrhythmias could be ablated without the use of fluoroscopy. Despite screening the appropriateness of patients prior to attempting FF ablation and excluding those in whom a transseptal or transbaffle puncture was anticipated, crossover to fluoroscopy use occurred in 24% of patients. The crossover was most often due to the need for left atrial access for the treatment of concealed left-sided accessory pathways and left-sided atrial tachycardia (identified in 30 of our cohort patients). Thus, the reasoning for fluoroscopy use was most dependent on diagnosis/arrhythmia substrate rather than on comorbidities.

EAM has previously been shown to reduce the amount of fluoroscopy used during ablation. Papagiannis et al. demonstrated a reduction of more than 50% in fluoroscopic time for supraventricular tachycardia (SVT) procedures in children using the EnSite™ NavX™ 3D EAM system (Abbott Laboratories, Chicago, IL, USA).^6^ Casella et al. showed further reduction in fluoroscopy time in the ablation of SVT in adults with an average of only two minutes of fluoroscopy use and several zero fluoroscopy-use cases, also using the EnSite™ NavX™ system (Abbott Laboratories, Chicago, IL, USA).^7^ Similarly, Razminia et al. described their experience using EAM in conjunction with intracardiac echocardiography to perform SVT, ventricular tachycardia, and atrial fibrillation ablations safely and effectively in adults without the use of fluoroscopy.^8^ Other individual case reports and series have described SVT or ventricular tachycardia ablations without the use of fluoroscopy in children and adults using 3D anatomical mapping systems, demonstrating the safety and efficacy of the procedure.^9–12^ In addition to these case series, several multicenter investigations have evaluated ablation outcomes, comparing procedures performed with minimal fluoroscopy and the use of EAM and regular fluoroscopy procedures. Giaccardi et al. completed a retrospective review of patients at three medical centers with attempted FF ablation and compared outcomes to patients with fluoroscopic-guided ablation procedures. While excluding cases requiring transseptal puncture, the study identified a high rate of performing the entire procedure without fluoroscopy (86%), with similar acute procedural success between those with (98%) and without (97%) fluoroscopy use and no difference in complication rates between the two groups.^13^ The investigators of the Spanish Multicenter Registry of FF Ablation prospectively investigated only FF products and identified a similarly high acute procedural success rate (89%) for predominantly right atrial and right ventricular arrhythmias, with a complication rate of 1.7%. Features associated with fluoroscopy use included the center performing the ablation and the ultimate success of the procedure.^14^ In contrast to these studies, the current study included arrhythmias located in the left and right atria, as well as idiopathic ventricular tachycardia. In addition, we investigated procedural characteristics associated with brief fluoroscopy use and identified the need for a transseptal puncture as the most common reason for fluoroscopy. However, even when left atrial access was required, 12 of the 30 transseptal puncture cases were done without fluoroscopy.

In addition to reducing radiation exposure to the patient, staff, and operator, performing FF ablation may allow the operator and/or laboratory staff to be free from lead protective garments for at least some, if not all, of the procedure. The potential impact of this is significant, since in two surveys of physicians who perform procedures in an interventional laboratory necessitating the use of such garments, nearly half had spine problems, as compared with 27% of the general population.^3,15^ Additionally, there is a linear relationship between the incidence of spine problems and the number of procedures performed in the fluoroscopy suite. Through ongoing use and refinement of FF ablation, we anticipate that a new generation of EP physicians and laboratory staff will benefit from fully using the innovative techniques that are, or will become, available and thus see a reduction in work-related orthopedic injuries.

### Limitations

This is a retrospective study and the proposed benefits are limited to hypothesis generation only. Despite a thorough review of medical records, it is possible that some IFF cases were missed due to documentation error. Further evaluation of this technique and its suspected long-term benefits will require a longitudinal sample of patients, staff members, and physicians as well as additional multicenter prospective studies investigating acute and long-term outcomes and complications with FF ablation. Due to the low number of patients with each arrhythmia, it was not felt that comparisons of fluoroscopy use within each arrhythmia type would be meaningful. Lastly, retrospective data collection from medical records is inherently limited by the quality of documentation.

## Conclusion

In this single-center study that includes both pediatric and adult patients referred for FF ablation, minimal fluoroscopy was required in 24% of cases. There were no differences in acute procedural success, catheter placement time, or complication rate between FF ablation or ablation with minimal fluoroscopy use. The most common reason for crossover in our cohort was the need for transseptal puncture, rather than patient comorbidities. Even with careful selection of patients for FF ablation, providers need to be prepared for the need for fluoroscopy use, particularly when the underlying diagnosis of the arrhythmia is unclear.

## Figures and Tables

**Table 1:** Patient Characteristics

Characteristics	All Cases (n = 138)
Male gender, n (%)	60 (43.5%)
Age, median (mean ± SD)	17.0 (25.0 ± 19.1) years
Height, median (mean ± SD)	167.3 (163.6 ± 18.1) cm*
Weight, median (mean ± SD)	63.7 (65.1 ± 23.0) kg
Body mass index	22.49 (23.6 ± 6.1) kg/m^2^*
Comorbidities	
Prior surgery	49
Complex congenital heart disease	13
Valve disorder	11
Coronary artery disease	0
Left ventricular dysfunction	0
Heart failure	1
Hypertension	10
Stroke	0
COPD or asthma	10
Diabetes	4
Obesity	30
Cases with comorbidities, n (%)	77 (55.8%)

n: number; SD: standard deviation; COPD: chronic obstructive pulmonary disorder.

*n = 137 (height not available for one patient).

**Table 2:** Crossover Patient Characteristics

Characteristics	Crossover Cases (n = 33)
Male gender, n (%)	18 (54.6%)
Age, median (mean ± SD)	16 (19.6 ± 16.0) years
Height, median (mean ± SD)	162.6 (158.8 ± 25.1) cm
Weight, median (mean ± SD)	59.0 (60.6 ± 7.22) kg
BMI	21.5 (23.0 ± 7.22) kg/m^2^
Comorbidities	
Prior surgery (noncardiac not involving head or limbs)	8
Complex congenital heart disease	0
Valve disorder	2
Coronary artery disease	0
Left ventricular dysfunction	0
Heart failure	0
Hypertension	1
Stroke	0
COPD or asthma	2
Diabetes	0
Obesity	9
Cases with comorbidities, n (%)	17 (55%)
Final diagnosis	
Atrioventricular nodal reentry tachycardia	7
Atrioventricular reentry tachycardia	17
Atrial tachycardia	5
Persistent junctional reciprocating tachycardia	2
Right ventricular outflow tract PVCs/VT	2
Atrial flutter	1
Junctional ectopic tachycardia	0
Mahaim pathway	0
Cases with transseptal puncture	18

n: number; SD: standard deviation; COPD: chronic obstructive pulmonary disorder; PVCs: premature ventricular complexes; VT: ventricular tachycardia.

**Table 3:** Repeat Patient Case Characteristics

Patient	First Procedure		Second Procedure		Third Procedure		Comorbidities*
	Final DX	Fluoroscopy	Final DX	Fluoroscopy	Final DX	Fluoroscopy	
1	AVNRT	No	PJRT	NO	PJRT	Yes	• None
2	AVRT	Yes	AVRT	NO	N/A	N/A	• None
3	AET	Yes	AET AFL	Yes	N/A	N/A	• Obesity
4	AVNRT	No	AVNRT	No	N/A	N/A	• None
5	AVNRT	No	Echo beats	No	AVNRT	No	• Obesity
6	AVNRT	No	AVNRT	No	N/A	N/A	• CHD • Obesity
7	AVNRT AVRT	No	None	No	N/A	N/A	• CPOD/asthma
8	PJRT	No	PJRT	No	PJRT	No	• CHD • Valve disorder
9	AVRT	Yes	AVRT	No	N/A	N/A	• None
10	AVNRT	No	AVNRT	No	N/A	N/A	• Obesity
11	AVNRT	No	AVNRT	No	N/A	N/A	• None

DX: diagnosis; AVNRT: atrioventricular nodal reentry tachycardia; AVRT: atrioventricular reentry tachycardia; AET: atrial ectopic tachycardia; PJRT: persistent junctional reciprocating tachycardia; AFL: atrial flutter; N/A: not applicable; CHD: congenital heart disease; COPD: chronic obstructive pulmonary disorder.

*First-procedure comorbidities were assumed to exist for second and third procedures as well where appropriate.
